# Anaplastic thyroid carcinoma: Clinicopathologic and immunohistochemical study of 144 cases with special emphasis on the spectrum of histologic features

**DOI:** 10.1007/s00428-026-04456-8

**Published:** 2026-02-27

**Authors:** David I. Suster, Ondrej Daum, Michael Michal, A. Craig Mackinnon, Vania Nose, Michal Michal, Saul Suster

**Affiliations:** 1 Rutgers New Jersey Medical School, University Hospital, 150 Bergen Street, Newark, NJ 07103 USA; 2 Charles University, Faculty of Medicine in Plzen, Plsen, Czech Republic; 3 University of Alabama, Birmingham, AL USA; 4 Massachusetts General Hospital and Harvard Medical School, Boston, MA USA; 5 Medical College of Wisconsin, Milwaukee, WI USA; 6 Bioptical Laboratory, Ltd., Plzen, Czech Republic; 7 University of Texas Southwestern, Dallas, TX, USA

**Keywords:** Thyroid anaplastic carcinoma, Undifferentiated carcinoma, Squamous differentiation, Immunohistochemistry, CD10, P40, Cytokeratin, PAX8, TTF1, Ki-67

## Abstract

We have studied 144 patients with anaplastic thyroid carcinoma (ATC) to evaluate the utility of a select panel of immunohistochemical stains for diagnosis and to better define their spectrum of histologic features. There were 97 women and 47 men, aged 48 to 103 years (mean: 67.8). The tumors were seen against a background of papillary thyroid carcinoma in 33 cases, follicular thyroid carcinoma in 28, poorly differentiated carcinoma in 5, and thyroid nodular disease in 31. Histologically, two basic patterns of growth were observed, one predominantly composed of spindle and pleomorphic cells, and one composed of round epithelioid cells. Several unusual morphologic features were also observed focally that can introduce difficulties for diagnosis, including tumors with squamous differentiation (47 cases; 32.6%), clear cells (17; 11.8%), paucicellular (17; 11.8%), inflammatory (16; 11%), pseudoangiosarcomatous (11; 7.6%), rhabdoid (9; 6.2%), and osteoclastic giant cells (8; 5.5%). Immunohistochemical staining was performed in 107 cases: AE1/AE3 cytokeratin stains were positive in 63 cases (58.8%) and CK8/18 in 46 (37.3%); PAX8 was positive in 35 cases (32.7%); TTF1 was only focally positive in 4 cases (3.7%); p40 was positive in 22 cases (20.5%), mostly in the areas of squamous differentiation. Strong CD10 positivity was also seen in 97 cases (90.6%). Our study shows that anaplastic carcinoma has the potential for simulating a large variety of tumors and underscores the difficulties for making the diagnosis using immunohistochemistry; cytokeratins, PAX8 and TTF1 were of limited utility and showed inconsistent results emphasizing the importance of clinicopathologic correlation for the diagnosis.

**Supplementary Information:**

The online version contains supplementary material available at 10.1007/s00428-026-04456-8.

## Introduction

Anaplastic thyroid carcinoma (ATC) is the rarest form of thyroid cancer and is associated with highly aggressive behavior. These tumors are characterized by great morphologic heterogeneity which under certain circumstances can introduce difficulties for the diagnosis. Traditionally, the prototypical form of ATC has been one that closely resembles a high grade pleomorphic and spindle cell sarcoma; however, additional and unusual morphologic presentations have been described that have come to further complicate the picture [1–4]. One of the most challenging aspects for the diagnosis of ATC has been the role of immunohistochemical staining. Despite being considered an epithelial malignancy derived from follicular cells, thyroid-associated and epithelial-associated markers have demonstrated restricted and variable expression in these tumors, making the immunohistochemical confirmation of the diagnosis problematic [5–11].

We have studied a series of 144 cases of ATC from 4 different institutions to better delineate the morphologic spectrum of these lesions and to assess in a large cohort the relative value of the most commonly employed immunohistochemical markers utilized for their diagnosis. The results of our study along with a review of the literature are presented.

## Materials and methods

Cases diagnosed as ATC or undifferentiated carcinoma of the thyroid were retrieved from the surgical pathology files of the Medical College of Wisconsin, Milwaukee, WI; Biopticka Laboratory, Plsen, Czech Republic; the University of Alabama at Birmingham, AL; the Massachusetts General Hospital and Harvard Medical School, Boston, MA; and from the personal consultation files of one of the authors over a 30-year period (1990–2020). Criteria for diagnosis were applied according to the World Health Organization (WHO) [12]. Only cases of total or subtotal resection were included in the study; core biopsies or incisional biopsies were censored from the study. From 5–16 representative hematoxylin and eosin (H&E) stained glass slides were available for review from each case. Representative paraffin blocks were available for immunohistochemical studies in 107 cases. Clinical information was obtained from the medical records; slides were reviewed by the contributing pathologists from the various institutions and centrally reviewed by two of the authors (DS, SS). The study was carried out under approved institutional review protocol (IRB) from the Medical College of Wisconsin.

Immunohistochemical staining was performed on whole tissue sections of formalin-fixed paraffin-embedded tissue samples using a standard technique. Immunohistochemical stains for all markers tested were performed centrally at a single laboratory (Wisconsin Diagnostic Labs, Milwaukee, WI). Briefly, immunohistochemical staining was performed using reagents from the Dako Envision FLEX kit and the Dako AutostainerPlus stainer (Agilent, Santa Clara, CA). Following pretreatment with Target Retrieval Solution, tissue was blocked with a peroxidase-blocking reagent for 5 min and incubated with the primary antibody at room temperature. Signals were detected using the Dako FLEX detection kit. Counterstaining was performed with Envision FLEX hematoxylin for 7 min at room temperature. Appropriate positive and negative controls were run concurrently for all antibodies tested. The primary antibodies included: AE1/AE3 (Dako, Agilent, prediluted; Dako, Santa Barbara); CK8/18 (Leica, Wetzlar, clone 5D3, prediluted); TTF1 (Dako, 8G7G3/1, 1:300); p40 (Biocare, polyclonal, 1:100, Concord, CA); Ki-67 (Clone MIB1, Agilent, Dako, prediluted); PAX8 (Proteintech, Rosemont, Il, polyclonal, prediluted); and CD10 (clone 56C6, Agilent, Dako, prediluted). Additionally, immunohistochemical stains for SMA, desmin, S100, CD31, CD34, and STAT6 were performed as part of the initial diagnostic work-up in selected cases. The immunohistochemical reaction was graded as positive based on nuclear, cytoplasmic or membrane reactivity for the various antibodies. Positivity was semi quantitatively graded as negative (0), focal/mild (up to 20% of tumor cells; +), moderate (20–50%; + +), or strong/diffuse (> 50%; +  + +). Ki-67 was graded by counting the percentage of positive nuclei per 100 cells in the most active areas (hot spots).

## Results

Detailed clinical and pathologic features of our patients are listed in supplemental Tables 1 and 2. In brief, there were 97 women and 47 men, aged 48 to 103 years (mean: 67.8). There were only 2 patients under 50 in the group and 76% of patients were over the age of 60. The tumors occurred in the left lobe in 63 patients, right lobe in 42, and the location could not be determined in 39. Presenting clinical symptoms included hoarseness, dysphagia, and a rapidly enlarging mass in the neck region. All patients in the study were treated by surgical excision; in 69 patients a complete surgical resection could be accomplished; in 75 only a partial or subtotal resection was done due to extensive compromise of adjacent structures. The tumors were described grossly as gray-white, firm, with areas of hemorrhage and necrosis. The tumors were associated with an underlying pathological abnormality of the thyroid in 99 cases. 33 cases showed an associated papillary thyroid carcinoma; 28 cases were associated with follicular thyroid carcinoma; 5 cases showed poorly differentiated thyroid carcinoma; 31 cases were associated with benign nodular thyroid disease; and 6 cases showed features of chronic lymphocytic thyroiditis. Patients presented with locoregional metastases to lymph nodes and skeletal muscle in the head and neck. Preoperative radiation and/or chemotherapy was given to 64 patients, and postoperative chemotherapy was administered to 35. Details of treatment other than surgery were not available for 45 patients. Clinical follow-up was only available for 63 patients; all died of their tumors between 2–20 months (mean: 11.5 months).

**Table 1:** Histologic features and underlying pathologic conditions in 144 patients with ATC

Histology	
Epithelioid	72 (50%)
Spindle/pleomorphic	47 (32.6%)
Mixed	25 (17.3%)
Squamoid	47 (32.6%)
Paucicellular	17 (11.8%)
Clear cell	17 (11.8%)
Inflammatory	15 (10.4%)
Pseudoangiosarcomatous	11 (7.6%)
Rhabdoid	9 (6.25%)
Osteoclastic	8 (5.5%)
Underlying Conditions	
Papillary thyroid carcinoma	33 (22.9%)
Follicular thyroid carcinoma	28 (19.4%)
Poorly differentiated carcinoma	5 (3.4%)
Nodular thyroid disease	31 (21.5%)
Chronic lymphocytic thyroiditis	6 (4.1%)

**Table 2:** Summary of immunohistochemical stains in 107 cases of ATC

Immunostain	Number and percentage of positive cases
TTF1	4 (3.7%)
PAX8	35 (32.7%)
AE1/AE3	63 (58.8%)
CAM5.2	40 (37.3%)
p40	22 (20.5%)
CD10	97 (90.6%)

### Histopathologic findings

The tumors were all characterized by their variegated histologic appearance (Table 1). Two basic cytomorphologic types could be identified: tumors predominantly composed of atypical spindle or pleomorphic cells resembling for the most part an undifferentiated pleomorphic sarcoma, and tumors composed of round, epithelioid cells with abundant cytoplasm more closely resembling an epithelial malignancy. Most tumors showed combinations in various proportions of these two constituents; however, in most cases one pattern usually predominated over the other.

#### Spindle/pleomorphic type

**Fig. 1:**
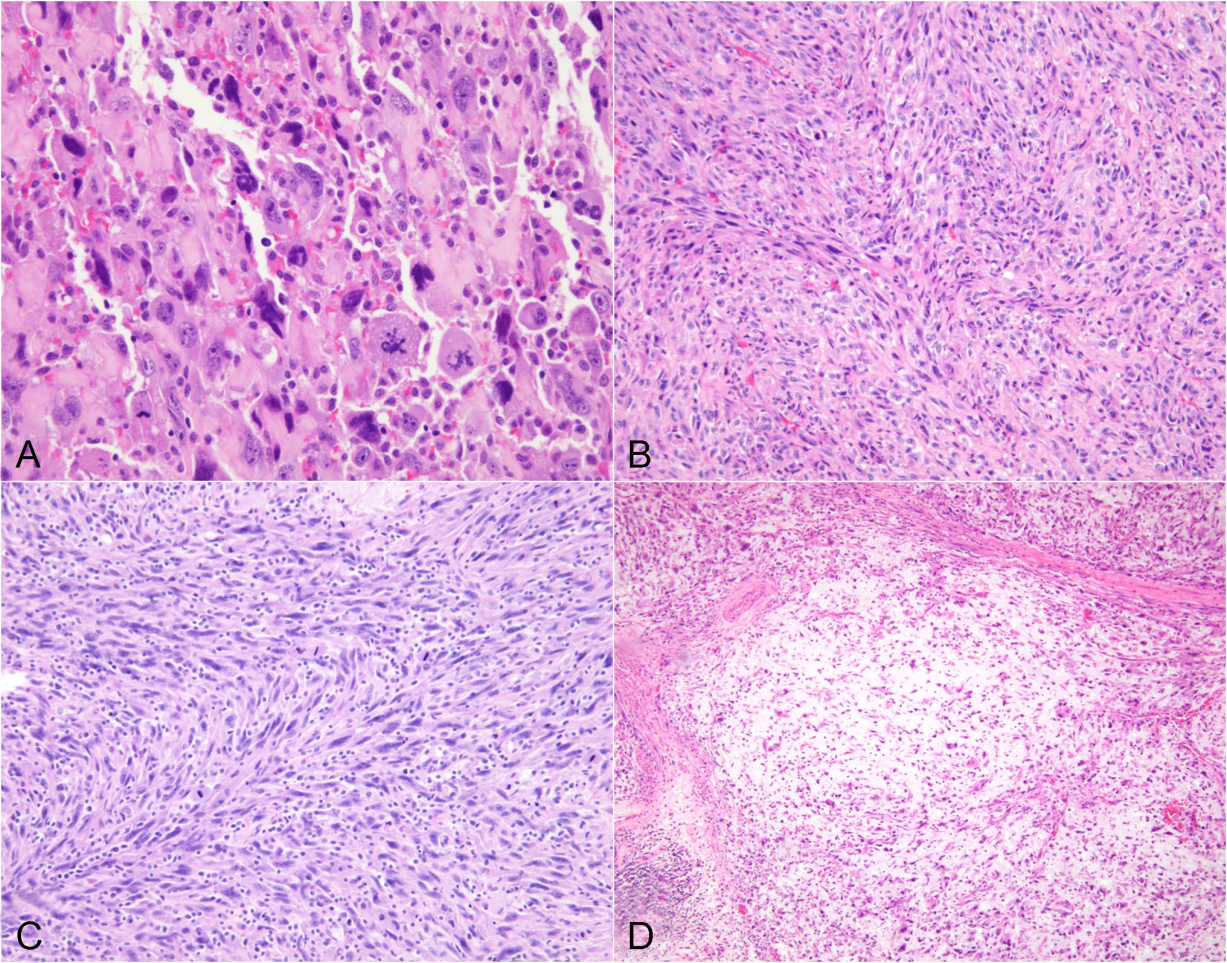
**A**) Spindle/pleomorphic ATC shows large, anaplastic tumor cells with enlarged, hyperchromatic nuclei and multinucleation, and abnormal mitotic figures; **B**) Spindle/pleomorphic ATC shows population of atypical spindle cells with storiform pattern; **C**) Spindle ATC with relatively bland-appearing tumor cells adopting a herringbone pattern reminiscent of fibrosarcoma; **D**) Spindle ATC with prominent stromal myxoid changes simulating low-grade myxofibrosarcoma.

Forty-seven cases were composed of a predominantly spindle/pleomorphic pattern of growth, defined as a tumor containing more than 70% of this growth pattern and composed of spindle and/or pleomorphic tumor cells. These tumors most closely resembled a spindle cell sarcoma and adopted two types of histologic appearances; one as a high-grade spindle/pleomorphic proliferation of tumor cells with marked cytologic atypia, bizarre multinucleated tumor cells, and frequent abnormal mitoses that closely resembled undifferentiated pleomorphic sarcoma (so-called “malignant fibrous histiocytoma” of the older literature) (34 cases), and a second form characterized by a low grade fascicular spindle cell proliferation more closely reminiscent of a low to intermediate grade fibrosarcoma or leiomyosarcoma (13 cases). The cases showing features of the spindle/pleomorphic type roughly corresponded to cases designated as “spindle and giant cell carcinoma” in the older literature. These tumors displayed fascicles of large, hyperchromatic spindle cells with enlarged nuclei, prominent nucleoli and eosinophilic cytoplasm admixed with larger, often multinucleated giant cells with marked cytologic atypia and bizarre nuclei (Fig. 1A). The tumor cells often adopted a storiform pattern and were associated with extensive areas of hemorrhage and necrosis (Fig. 1B). Mitotic activity was high (5 to > 10 per 10 high power fields) and vascular invasion, particularly of mid- and large caliber vessels was prominent. Many of the tumors also showed areas of stromal sclerosis with entrapment of isolated small cell clusters or individual tumor cells, as well as foci of perineural invasion. The second pattern of growth observed in these tumors was characterized by a relatively bland, low-grade fascicular spindle cell proliferation with minimal to moderate cytologic atypia, absence of significant nuclear pleomorphism or multinucleation, and low mitotic activity (1–4 mitoses per 10 high power fields). The tumors often adopted a fascicular or herringbone growth pattern or showed alternating fascicles of spindle cells cut at right angles simulating a smooth muscle tumor (Fig. 1C). Some cases displayed prominent myxoid stromal changes reminiscent of low-grade myxofibrosarcoma (Fig. 1D). Two cases contained foci of osseous metaplasia.

#### Epithelioid type

**Fig. 2:**
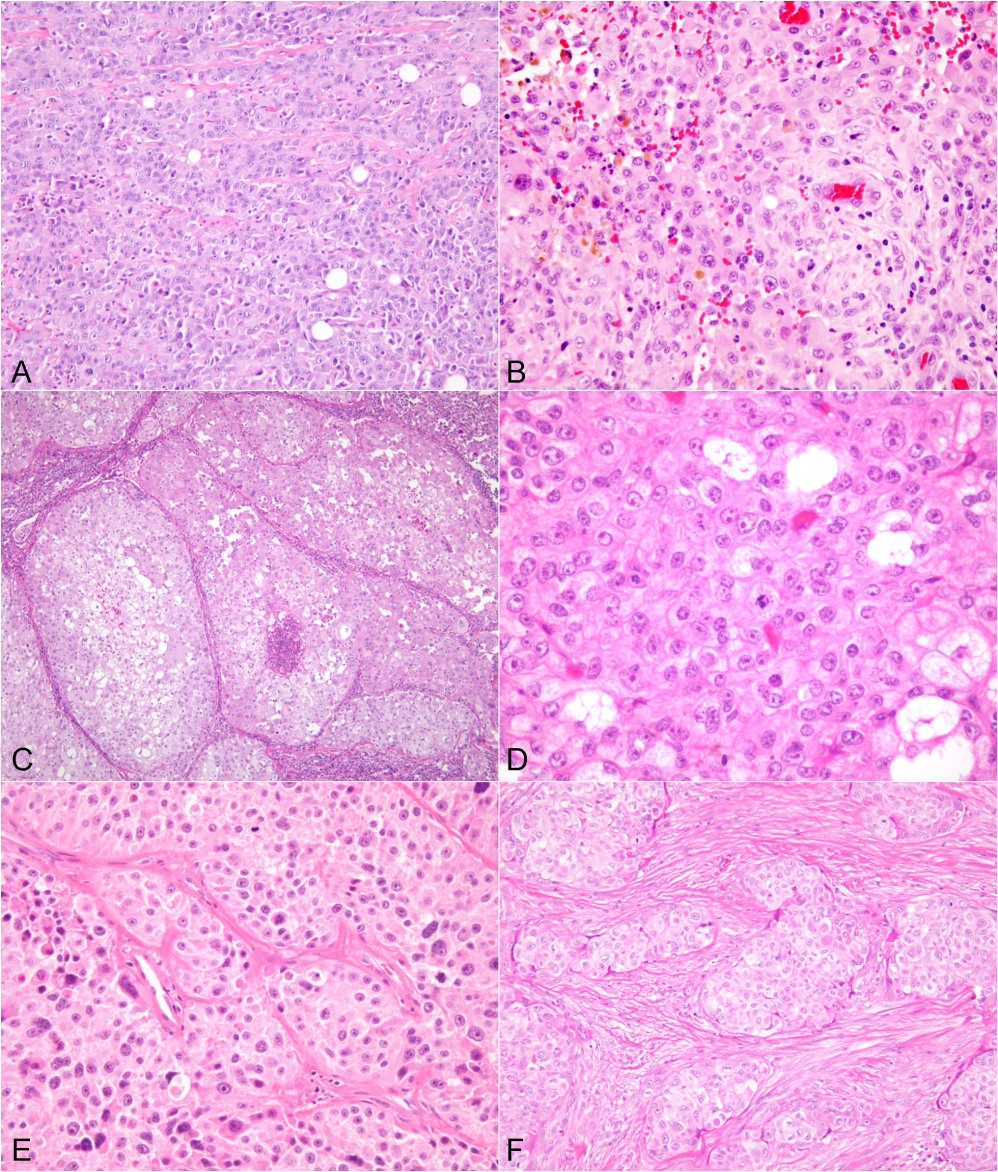
**A**) Epithelioid ATC: scanning magnification shows sheets of monotonous round cells with abundant cytoplasm with minimal intervening stroma; **B**) Higher magnification of epithelioid ATC shows sheets of round to polygonal epithelioid cells with enlarged nuclei and prominent nucleoli and occasional mitotic figures; **C**) Epithelioid ATC showing nodular pattern of growth with large, sharply delineated nodules with central areas of comedonecrosis; **D**) High power view of epithelioid ATC shows monotonous round to polygonal tumor cells with enlarged nuclei and scattered mitoses. **E**) Epithelioid ATC with a nested growth pattern is shown bearing a vague resemblance to melanoma. **F**) Another example of epithelioid ATC with a nested growth pattern and prominent intervening fibrotic stroma

Seventy-two cases were of epithelioid type and were characterized by a predominant population of large round to polygonal tumor cells with abundant cytoplasm comprising more than 70% of the tumor. These tumors also showed some variegation in growth patterns. The most common growth pattern observed was characterized by sheets of relatively monotonous epithelioid cells with mild to moderate cytologic atypia, mild nuclear pleomorphism, and scattered mitotic figures (Fig. 2A, B). All cases were associated with extensive areas of tumor cell necrosis, vascular invasion, and infiltration of surrounding tissues. In some cases, areas showing marked cytologic atypia of the epithelioid cells with scattered multinucleated cells and abnormal mitoses could also be seen. A second pattern of growth observed in 12 of these tumors consisted of a multinodular process made up of large solid nodules of tumor cells separated by fibrovascular stroma with focal central areas of necrosis (Fig. 2C). The tumor cells in these cases were also round, epithelioid or polygonal, with minimal variability and with varying degrees of cytologic atypia and mitotic activity (Fig. 2D). A less commonly observed pattern found in 6 cases was one characterized by small nests of tumor cells separated by desmoplastic stroma reminiscent of metastatic melanoma which contained enlarged and atypical tumor cells with hyperchromatic nuclei, prominent nucleoli, and scattered mitoses (Fig. 2E, F). Four cases showed associated foci of osseous metaplasia and 1 case showed extensive cartilaginous metaplasia. The metaplastic elements did not display any evidence of cytologic atypia that would warrant a diagnosis of osteo- or chondrosarcoma. Vascular and perineural invasion were often encountered in these tumors.

#### Mixed spindle and epithelioid

Twenty-five cases showed an approximately equal admixture of spindle and epithelioid cells. Most were composed of sheets of intimately admixed spindle and epithelioid cells displaying marked cytologic atypia, mitotic activity with abnormal mitoses, with areas of hemorrhage and necrosis. Vascular and perineural invasion was often identified.

### Unusual histologic features

A significant number of cases in the study (125 cases) displayed unusual histological features, regardless of the predominant growth pattern. In most cases, the variant morphology was focal and gradually merged with other areas of more conventional spindle/pleomorphic or epithelioid anaplastic carcinoma; however, some cases were predominantly composed of the variant morphology.

#### Squamoid anaplastic carcinoma

**Fig. 3:**
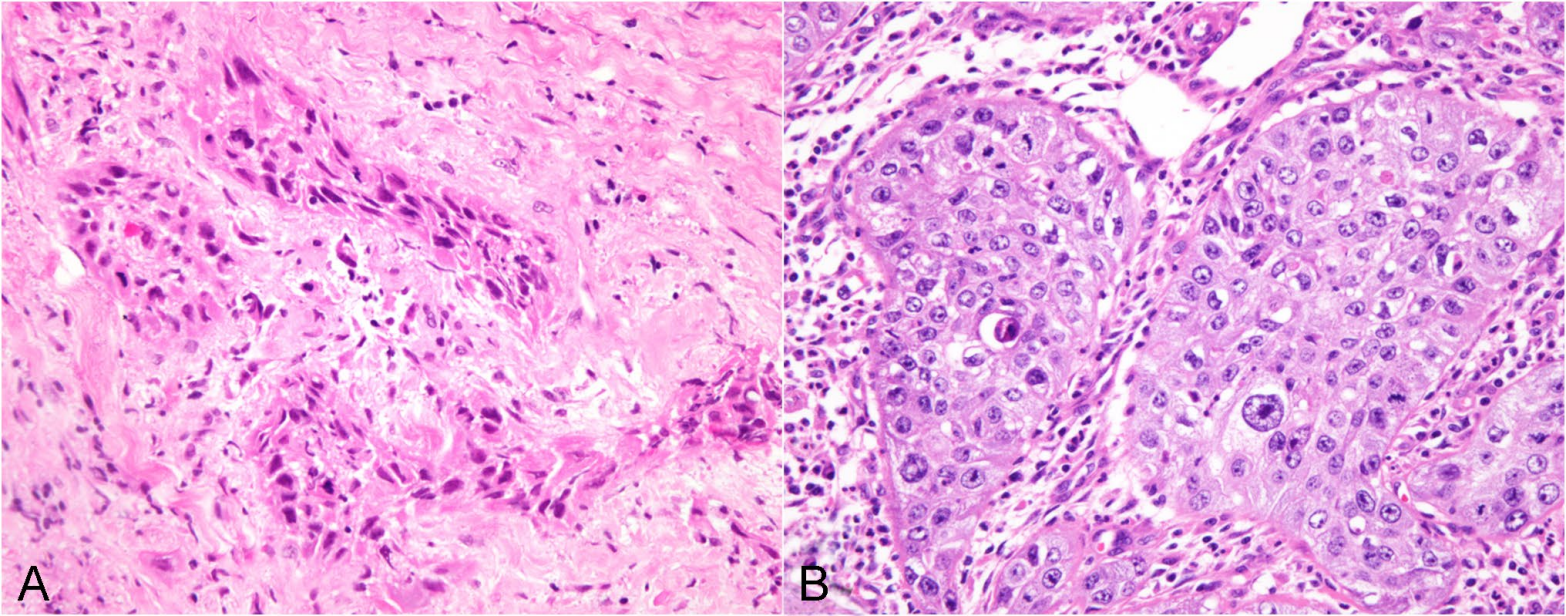
**A**) ATC with squamous differentiation showing irregular foci of poorly differentiated squamous cell carcinoma; **B**) ATC with squamous differentiation showing islands of well to moderately differentiated squamous cell carcinoma

47 tumors showed features of squamous differentiation. These areas were focal in 30 cases and extensive in 17; however, in no case was the tumor composed exclusively of squamous elements. The areas of squamous differentiation ranged from well-differentiated, keratinizing squamous cell carcinoma, to poorly differentiated non-keratinizing squamous cell carcinoma (Fig. 3A-B). Most of the tumors with squamous differentiation were primarily of epithelioid type, with subtle transitions between the sheets or lobules of epithelioid cells and the squamoid elements being observed. Transitions with spindle/pleomorphic areas could also be demonstrated.

#### Clear cell anaplastic carcinoma

**Fig. 4:**
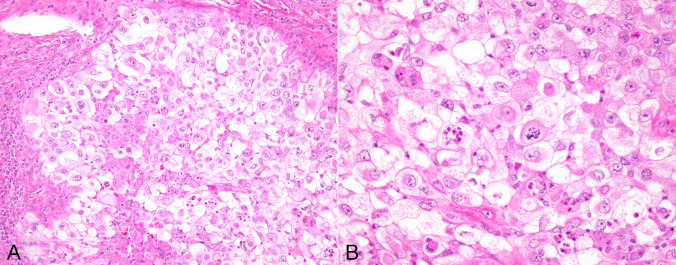
**A**) Clear cell variant of ATC shows lobules composed of tumor cells with abundant clear cytoplasm; **B**) Higher magnification from clear cell ATC shows population of round to polygonal tumor cells with abundant clear cytoplasm; notice abnormal mitosis

17 cases were remarkable for prominent cytoplasmic clearing of the tumor cells. Clear cell changes were most often seen in cases with epithelioid morphology and were present in both the sheet-like and the nodular pattern of growth (Fig. 4A, B). In 2 cases, clear cell changes were present in areas of squamous differentiation. The clear cell changes could be traced back to residual follicular structures in 4 cases of follicular carcinoma showing transformation to anaplastic carcinoma.

####  “Inflammatory” anaplastic carcinoma

**Fig. 5:**
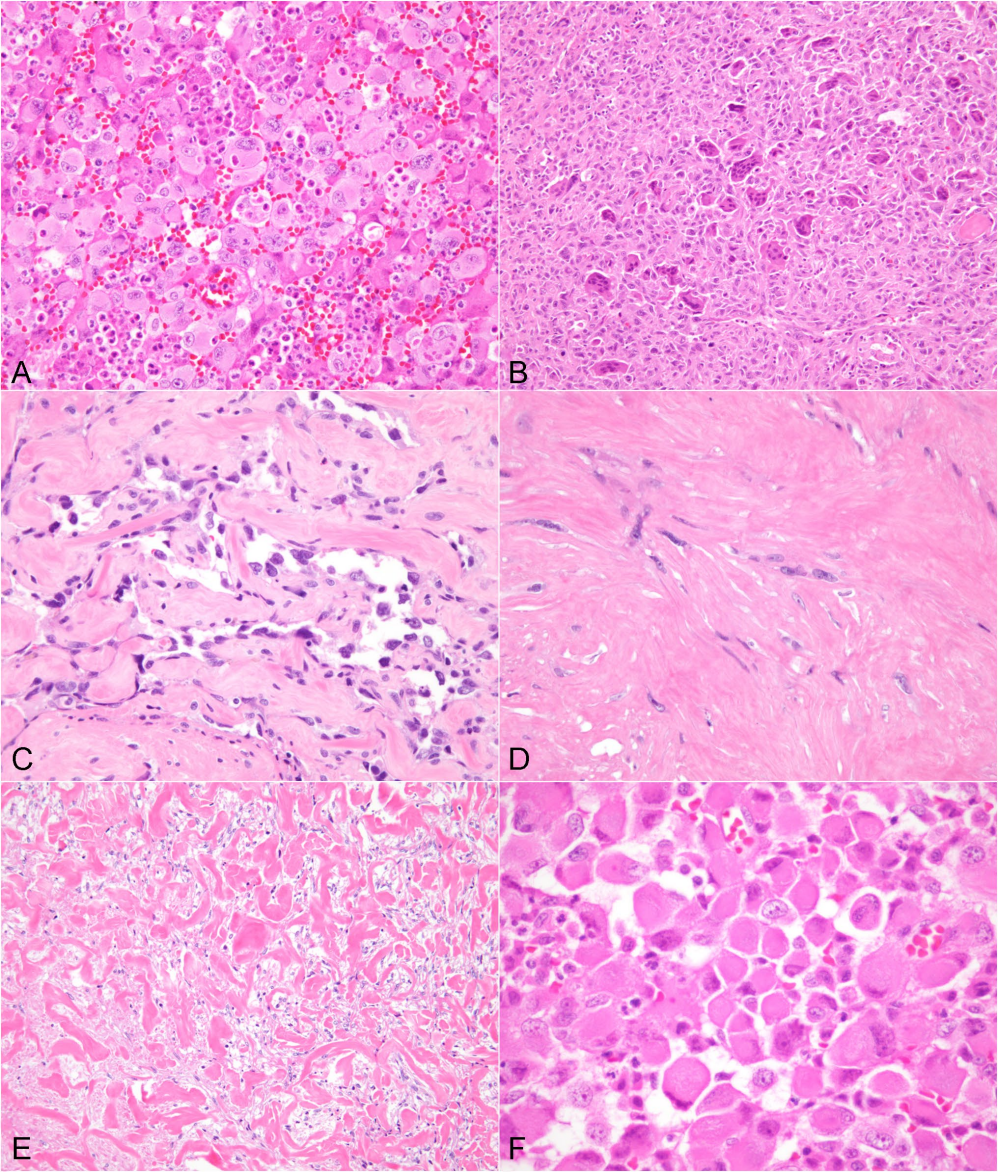
**A**) “Inflammatory” type of ATC shows discohesive population of large epithelioid tumor cells surrounded by abundant inflammatory cell infiltrate; **B**) Osteoclast-rich ATC shows areas containing abundant osteoclastic-type multinucleated giant cells surrounded by atypical spindle cell elements; **C**) Pseudoangiomatous pattern in ATC shows irregular vessel-like spaces lined by atypical cells resembling angiosarcoma. **D**) “Paucicellular” variant of ATC shows few scattered spindle cells with minimal cytologic atypia embedded in abundant collagenized stroma; **E**) ATC with prominent stromal hyalinization shows strands of keloidal collagen flanking small round and spindle cells reminiscent of a solitary fibrous tumor; **F**) ATC with “rhabdoid” component shows sheets of large epithelioid cells with prominent dense eosinophilic intracytoplasmic inclusions

15 cases showed features that were characterized by sheets of discohesive large tumor cells admixed with a heavy neutrophilic infiltrate in the stroma resulting in a striking histologic appearance similar to that seen in some poorly differentiated carcinomas arising from other organs. The tumor cells in these cases were large, epithelioid, and displayed marked cytologic atypia and mitotic activity (Fig. 5A). Most of these cases showed areas of transition with more conventional epithelioid elements, although in 2 cases the inflammatory areas were associated with spindle cell elements.

#### Osteoclast-rich anaplastic carcinoma

8 cases were characterized by large areas containing an abundance of osteoclastic multinucleated giant cells reminiscent of giant cell tumor of bone. The osteoclastic giant cells contained numerous benign-appearing nuclei and abundant cytoplasm and were surrounded by the atypical epithelioid or spindle tumor cells (Fig. 5B).

#### Pseudoangiosarcomatous anaplastic carcinoma

11 cases were characterized by focal areas that resembled anastomosing vascular channels simulating an angiosarcoma. The vessel-like spaces were lined by enlarged highly atypical cells and most likely resulted from tissue breakdown due to loss of cohesion of islands of anaplastic tumor cells entrapped within sclerotic stroma (Fig. 5C). This feature was most commonly seen in tumors with a predominant epithelioid appearance and in tumors with squamous features.

#### Paucicellular anaplastic carcinoma

17 cases were characterized by broad areas showing a sparse population of spindle to oval cells with minimal atypia and only rare mitoses embedded in abundant sclerotic stroma (Fig. 5D). The lesions resembled fibromatosis or Riedel’s thyroiditis, and in a few of the cases they were also reminiscent of solitary fibrous tumor due to the linear deposition of rope-like collagen flanking the bland spindle cells (Fig. 5E). The paucicellular areas were focal in 4 and extensive in 13 cases (> 50% of the lesion). 5 cases showed moderate cytologic atypia of the spindle cells with occasional scattered mitotic figures. Correct diagnosis was accomplished by identifying the more traditional spindle or epithelioid cell areas displaying more pronounced features of cytologic atypia, and due to the widely invasive nature of the tumors.

#### Rhabdoid anaplastic carcinoma

9 cases displayed sheets of cells with rhabdoid cytoplasmic inclusions (Fig. 5F). The rhabdoid cells in all cases were associated with tumors predominantly composed of epithelioid cells. The rhabdoid areas were usually focal and involved 20–30% of the tumor.

### Immunohistochemical findings

The results of immunohistochemical staining in our cases are summarized in Table 2.

#### Cytokeratin stains

**Fig. 6:**
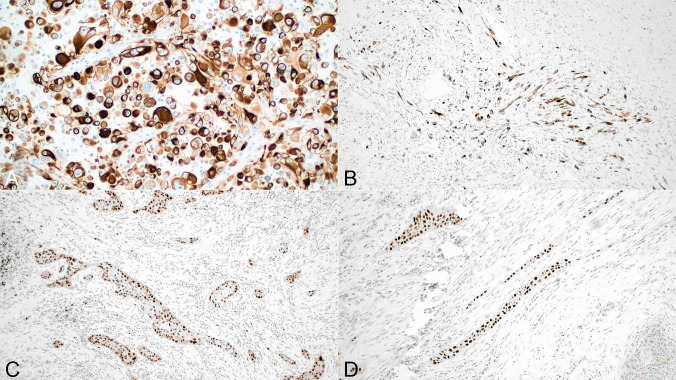
**A**) Immunohistochemical staining for cytokeratin AE1/AE3 in epithelioid variant of ATC shows strong cytoplasmic staining of the tumor cells; **B**) Focal staining for cytokeratin AE1/AE3 is seen in this example of spindle ATC; **C**) Nuclear positivity for p40 is seen in focus of squamous differentiation in ATC; **D**) Thin strands of small tumor cells show strong nuclear positivity for p40 in this ATC with squamous differentiation; these poorly differentiated areas were not readily recognizable as squamous on H&E

AE1/AE3 cytokeratin and CK8/18 were positive in 63 (58.8%) and 40 cases (37.3%), respectively. While AE1/AE3 seemed to be more sensitive for identifying tumor cells than CK8/18, both showed great variability in the staining pattern ranging from very focal and weak (in 32 cases for AE1/AE3 and 31 for CK8/18) to strong and diffuse (in 31cases for AE1/AE3 and 10 cases for CK8/18). Both keratin stains were strongly positive in the squamous or squamoid elements in all the tumors. The tumors that stained strongly for keratins were mostly of the epithelioid type; keratin staining with both antibodies was usually patchy and focal in the tumors composed of spindle and pleomorphic cells (Fig. 6A, B).

#### TTF1 and PAX8

Only 4 cases showed very focal staining for TT1, usually in areas transitioning with well-differentiated thyroid carcinoma components. PAX8 showed nuclear positivity in 35 cases (32.7%). The cases showing the strongest positivity were those containing areas of squamous differentiation, although 10 cases with predominant epithelioid morphology and absence of squamous elements were also strongly positive (Fig. 6C). In a few cases, some of the spindle cell areas also showed focal scattered positive cells.

#### P40

22 cases were positive for p40 (20.5%). The positive cases all corresponded to tumors displaying obvious features of squamous differentiation. In 5 cases, however, poorly differentiated areas that were not immediately recognized as squamous were also positive for this antibody (Fig. 6D). In general, the more conventional epithelioid and spindle cell components of the tumors were negative for this marker.

#### CD10

**Fig. 7:**
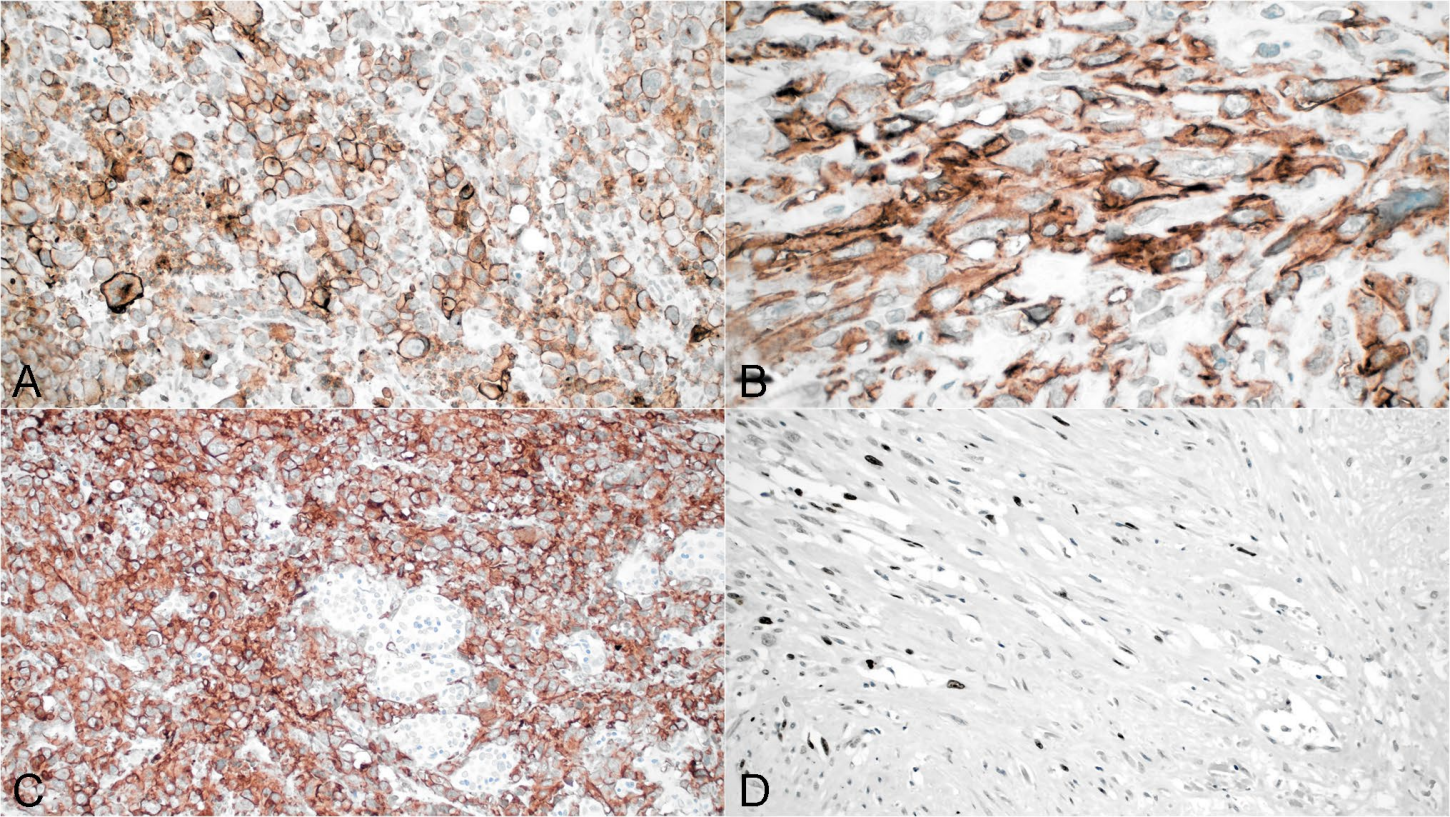
**A**) CD10 positivity is seen in this example of epithelioid ATC; **B**) CD10 positivity in spindle/pleomorphic ATC; **C**) CD10 positivity in epithelioid cells of ATC, notice negative staining of entrapped thyroid follicles; **D**) Paucicellular area in ATC shows scattered bland-appearing spindle cell nuclei that are positive for Ki-67

CD10 showed strong membranous and cytoplasmic positivity of the tumor cells in 97 cases (90.6%). The staining was equally strong in both the epithelioid and the spindle cell components (Fig. 7A, B). Residual normal, hyperplastic, or neoplastic follicular thyroid elements were uniformly negative (Fig. 7C).

#### MIB1

Nuclear positivity for Ki-67 ranged from 20–80% in 93 cases tested. There was no particular distribution associated with either spindle or epithelioid variants for this marker. An interesting finding was nuclear positivity seen in cases with paucicellular features which can be potentially helpful to differentiate the tumor cells from entrapped stromal fibroblasts (Fig. 7D).

## Discussion

Anaplastic thyroid carcinoma is a rare type of thyroid cancer characterized by undifferentiated histology and highly aggressive behavior. It presents clinically as a firm, rapidly enlarging mass in the neck of adult patients, often with a history of longstanding thyroid goiter. The histological appearance of this tumor can be highly variable but has traditionally been associated with a close resemblance to undifferentiated pleomorphic soft tissue sarcomas. The terminology for these tumors has evolved over the years, being initially regarded as undifferentiated carcinomas, but with the designation of anaplastic carcinoma being formally adopted by the World Health Organization (WHO) in 2017 [13]. Numerous studies have been published attempting to map out the histological spectrum of these tumors. Studies in the older literature referred to these tumors as “spindle and giant cell carcinoma” [14–16], although tumors composed of epithelioid cells, sometimes referred to as of “medullary” type, were also mentioned in the literature [14]. The 2017 edition of the WHO classification of thyroid tumors [13] recognized 3 main patterns of growth for anaplastic carcinoma: sarcomatoid (spindle cell), giant cell, and epithelioid, with the latter being defined as tumors showing squamoid or squamous differentiation. The latest WHO classification of thyroid tumors [15] has expanded this definition indicating that these tumors may be epithelioid, spindle, giant cell, paucicellular, rhabdoid, angiomatoid, or squamous, and may show metaplastic chondroid or osteoid features, often with a neutrophilic infiltrate. This variegation in morphology can often lead in practice to difficulties in diagnosis, particularly with small biopsies or when the clinical context is not clear [1, 3, 17].

Immunohistochemistry has been applied extensively for the study of anaplastic thyroid carcinoma. The results of different studies have shown wide variability with widely divergent results for the different markers [5–11]. In general, however, the value of immunohistochemistry has been rather limited for the diagnosis of these tumors.

We have presented a multi-institutional study from 4 tertiary care centers on 144 patients with anaplastic thyroid carcinoma to better clarify the spectrum of histopathologic features of these tumors and to assess the utility of a select panel of immunohistochemical markers for diagnosis. Our study showed that although these tumors are characterized by great variegation in histologic and cytologic appearances, there appear to be some consistent cytoarchitectural patterns that predominate. Traditionally, the prototypical histologic appearance of ATC has been that of a tumor that resembles a spindle and/or pleomorphic sarcoma. In recent years, tumors displaying obvious features of squamous differentiation have also been increasingly recognized as members of the family of ATC [12]. ATC displaying a predominantly epithelioid morphology, however, has not been as widely recognized and can be easily confused for metastases from other neoplasms.

Few studies addressing the histological features of ATC in large series of cases have been reported in recent years, with most of the current literature representing retrospective reviews on the topic [1–4]. The most comprehensive study so far on this tumor was by Xu et al. [18] who studied 227 resection specimens and 133 cytology/core biopsy samples and found that the most common growth pattern in these tumors was the spindle cell pattern (26%), followed by pleomorphic (23%), squamous (21%), epithelioid (19%), rhabdoid (8%), and osteoclastic (3%). In our study, we found that the most common morphologic pattern of ATC found in resection specimens from our patients was one composed predominantly of sheets of monotonous epithelioid cells. This epithelioid pattern of growth in ATC can be a source of confusion, particularly on small core or incisional biopsies in which the findings can be mistaken for metastases to the thyroid from other types of epithelioid malignancies. Because of the round to polygonal shape of the tumor cells, the well-defined cell borders, and the abundant cytoplasm, the epithelioid type of ATC has been previously regarded by some as being synonymous with squamoid or squamous ATC [3, 4, 18, 19]; however, most epithelioid ATCs do not display true features of squamous differentiation (i.e., intercellular bridges, keratinization) and should be distinguished from tumors which do. The epithelioid tumors in our study frequently presented as sheets of monotonous epithelioid cells with variable cytologic atypia and with extensive areas of necrosis merging with residual hyperplastic follicular elements or remnants of differentiated thyroid malignancy.

Another interesting aspect of our study is that it allowed us to assess the relative frequency of unusual growth patterns associated with ATC. In our series, we found that squamous differentiation (sometimes designated as squamoid features) was a very common finding in these tumors (32.6%), with a much higher incidence than previously reported in the literature [4, 20]. It is important to note that the squamous or squamoid features were associated in all cases with other areas of conventional spindle or epithelioid elements in the tumor and in many cases were quite focal. The high incidence of squamous differentiation in these tumors should raise the suspicion of ATC in any tumor biopsy from the thyroid region showing a high-grade tumor with features suggestive of squamous differentiation. The rare cases of thyroid tumors composed exclusively of squamous cell carcinoma elements are now regarded as equivalent to ATC on account of their similar behavior [12].

Other less common and unusual patterns of growth were also seen in our cases in smaller proportions, including tumors with a paucicellular appearance, previously reported as the “paucicellular variant” of ATC [1, 21]. Another unusual morphologic appearance of ATC observed in 17 of our cases was characterized by sheets of cells with clear cytoplasm. In most cases these tumors were associated with areas showing the features of conventional epithelioid ATC. Such cases need to be distinguished from metastases of clear cell tumors from other organs, particularly from the kidney. Sixteen cases were characterized by a dense inflammatory cell infiltrate in the stroma resulting in a discohesive population of atypical cells resembling tumors arising from the lung and other organs [22]. Another unusual morphologic appearance observed in our cases was one characterized by the formation of irregular anastomosing vessel-like spaces lined by atypical cells closely resembling angiosarcoma. Pseudoangiosarcomatous changes have been well described in the literature in ATC and are believed to be due to acantholytic changes in areas harboring poorly differentiated squamous cell carcinoma elements [16]. The pseudoangiosarcomatous changes in our cases were all focal and admixed with more conventional areas of ATC, but in small biopsy samples, the lesions can be confused for angiosarcoma [23]. Nine cases in our study (6.2%) also contained areas with tumor cells displaying a striking “rhabdoid” morphology, a feature that has been previously described in ATC [18, 24–26]. Given that rhabdoid change is usually a focal phenomenon in these tumors, they should not cause major difficulties for diagnosis after identifying the more conventional areas of ATC. Finally, ATCs containing a prominent component of osteoclastic giant cells accounted for only 5.5% of our cases. Tumors with these features have been consistently recognized in the literature but their incidence is unknown [16, 18]. The areas harboring the osteoclastic giant cells were usually focal and admixed with more conventional areas of ATC making this finding more of a curiosity than a diagnostic challenge.

The role of immunohistochemistry in the diagnosis of ATC has been controversial. Many efforts have been expended to identify specific and sensitive markers for this diagnosis but for the most part, most of them are either not widely available or have not been thoroughly validated [26–31]. Additionally, ATC can express several divergent lineage markers that can cause diagnostic confusion [32]. BRAF V600E mutations have been demonstrated by immunohistochemistry in about 38% of cases of ATC, particularly in cases showing squamous differentiation [18].

The role of BRAF in ATC has acquired more importance in recent years as a potential marker for targeted therapy; recent clinical trials with two selective BRAF inhibitors, Vemurafenib and Dabrafenib, have demonstrated overall increased response rates and substantial survival benefit with median overall survival of 43 months vs. the historical 5 months [33, 34]. BRAF testing may therefore acquire an increasingly significant role in the evaluation of ATC [35].

Traditionally, the diagnosis of ATC has been aided by identifying a precursor lesion in association with a higher-grade undifferentiated tumor or based on the characteristic clinical presentation and absence of a history of tumor elsewhere. In some instances, however, such as with small biopsy samples or when confronted with unusual morphologies, immunohistochemical staining has been utilized to assist with the diagnosis. Unfortunately, the use of various epithelial markers has been found to be of limited value for supporting the diagnosis of ATC. In general, reactivity for cytokeratins has been variable and inconsistent [36–39]. Our study showed at least focal keratin positivity in up to 58.8% of cases using AE1/AE3 and 37.3% using CK8/18. The prevalence of focal, weak and patchy staining for these markers may require the staining of several blocks in some cases for properly identifying the positive cells.

In our study, only 4 cases showed focal positivity for TTF1. A more recently developed marker with good specificity for thyroid follicular cell-derived neoplasms is PAX8. Initial studies appeared to suggest that PAX8 is a reliable and sensitive marker for the diagnosis of ATC [40, 41]; however, more recent studies have found a somewhat lower level of expression for this marker when using a monoclonal antibody [42]. In our study, staining for PAX8 using a polyclonal antibody was significantly lower than that cited in the literature; our cases were positive in only 32.7% of cases, and in almost half of them the staining was only focal and patchy. Due to the high incidence of squamous features in our cohort, we also included p40 in our panel of stains; 22 cases (20.5%) were positive for p40, mostly in cases showing obvious squamous differentiation, although 5 cases without obvious squamous areas also showed focal positivity. In a subset of cases, double labeling for PAX8 and p40 could be observed in the squamoid areas. An interesting finding in our study was strong positive staining in 97 cases (90.6%) for CD10. CD10 is a transmembrane metalloendopeptidase that is present in a wide variety of cell types and can also be expressed in some types of spindle cell sarcomas and sarcomatoid carcinomas [43–46]. Recently, Nazakawa et al. [47] reported high expression of CD10 in ATC; they found moderate to strong expression of this marker in all 47 cases they studied. Our study parallels those findings; we observed moderate to strong expression of CD10 in 90.6% of our cases across the entire spectrum of ATC. This finding is of interest given that in the appropriate clinical and pathologic context, the finding of strong CD10 positivity may be of help to support a diagnosis of ATC; however, the most important reason for awareness of this finding is in the setting of a metastasis from sarcomatoid renal cell carcinoma to the thyroid, which is also known to express CD10 [48] and can resemble ATC. Given that sarcomatoid renal cell carcinoma can also express CD10, cytokeratins, and PAX8, anaplastic tumors bearing this constellation of markers can easily be mistaken for metastasis from renal cell cancer. Clinical correlation and careful clinical history are therefore always indicated to rule out a kidney tumor in patients with ATC showing strong CD10, PAX8, and keratin positivity. Although some spindle cells sarcomas have also been found to express CD10 [49], soft tissue sarcomas rarely metastasize to the thyroid. In the context of a poorly differentiated sarcomatoid thyroid tumor, CD10 positivity is therefore more likely to correspond to an ATC, especially in the absence of any tumor elsewhere. This finding can be particularly helpful in small, biopsy samples where the absence of other markers, including cytokeratin and PAX8, may further obscure the diagnosis. CD10 can also be helpful in separating anaplastic elements within the tumor from other well-differentiated elements given that this marker is negative in normal follicular cells [47]. Finally, a not unexpected finding in our study was the demonstration of high proliferative activity using Ki-67 antibodies. Proliferative activity in our cases ranged from 20 to 80% nuclear positivity (mean: 45.3) in 93 cases tested, which is in-keeping with the published literature [4, 50]. Nuclear positivity was observed in the cells of the epithelioid cases as well as in tumors with paucicellular features, a finding that may be helpful for separating paucicellular ATC from fibromatosis or Riedel’s struma in small biopsy samples.

Our study also confirms the association of ATC with putative precursor or better-differentiated thyroid neoplasms; 33 cases arose in association with a papillary thyroid carcinoma (12 of follicular variant, 11 conventional, 9 oncocytic, and 1 tall cell); 28 cases in association with follicular thyroid carcinoma (16 conventional and 12 oncocytic); 5 with poorly differentiated thyroid carcinoma, and 31 cases against the background of nodular thyroid disease. Six cases also showed evidence of chronic lymphocytic thyroiditis in the uninvolved thyroid.

Some limitations of this study include the inclusion of cases from several separate institutions where sampling and processing techniques may differ and which could affect the results of the immunohistochemical stains or the incidence for the identification of the various unusual morphologic patterns seen in these tumors; the broad time range of the specimens analyzed in which variations in pre-analytical factors can contribute to affect the results of immunohistochemical staining; the limitation of the number of immunohistochemical stains used in the study; and the lack of molecular analysis of the cases in the study.

In short, we have examined 144 cases of ATC and found that they can exhibit a wide range of morphologic appearances. This study identified epithelioid morphology as the most common type of histology in these tumors, and squamous differentiation as a relatively common occurrence. Our immunohistochemical study confirmed that ATC shows only patchy and inconsistent cytokeratin positivity in approximately half of the cases, and that more specific thyroid-associated markers such as TTF1 and PAX8 are also of limited utility, emphasizing the importance of clinicopathologic correlation for the diagnosis.

## Supplementary Information

Below is the link to the electronic supplementary material.

Supplementary file1 (DOCX 14 KB)

Supplementary file2 (DOCX 55 KB)

## Data Availability

The data used in the creation of this article is not freely available, but is available upon reasonable request.

## References

[1] Baloch ZW, LiVolsi VA (2018) Special types of thyroid carcinoma. Histopathology 72:40–52 29239042 10.1111/his.13348

[2] Abe I, Lam AK-L (2021) Anaplastic thyroid carcinoma: updates on WHO classification, clinicopathological features and staging. Histol Histopathol 36:239–248 33170501 10.14670/HH-18-277

[3] Yang J, Barletta JA (2020) Anaplastic thyroid carcinoma. Semin Diagn Pathol 37:248–256 32624319 10.1053/j.semdp.2020.06.005

[4] Xu B, Ghossein RA (2023) Advances in thyroid pathology: high grade follicular c ell-derived thyroid carcinoma and anaplastic carcinoma. Adv Anat Pathol 30:3–10 36306188 10.1097/PAP.0000000000000380

[5] Wilson NW, Pambakian H, Richardson TC, Stokoe MR, Makin CA, Heyderman E (1986) Epithelial markers in thyroid carcinoma: and immunoperoxidase study. Histopathology 10:815–829 2428725 10.1111/j.1365-2559.1986.tb02580.x

[6] Hurlimann J, Gardiol D, Scazziga B (1987) Immunohistology of anaplastic thyroid carcinoma. A study of 43 cases. Histopathology 11:567–580 2442086 10.1111/j.1365-2559.1987.tb02667.x

[7] Ordonez NG, El-Naggar AK, Hickey RC, Samman NA (1991) Anaplastic thyroid carcinoma. Immunocytochemical study of 32 cases. Am J Clin Pathol 96:15–24 1712540 10.1093/ajcp/96.1.15

[8] Miettinen M, Franssila KO (2000) Variable expression of keratins and nearly uniform lack of thyroid transcription factor 1 in thyroid anaplastic carcinoma. Hum Pathol 31:1139–1145 11014583 10.1053/hupa.2000.16667

[9] Nonaka D, Tang Y, Chiriboga L, Rivera M, Ghossein R (2008) Diagnostic utility of thyroid transcription factors Pax8 and TTF2 (FoxE1) in thyroid epithelial neoplasms. Mod Pathol 21:192–200 18084247 10.1038/modpathol.3801002

[10] Bishop JA, Sharma R, Westra WH (2011) PAX8 immunostaining of anaplastic thyroid carcinoma: a reliable means of discerning thyroid origin for undifferentiated tumors of the head and neck. Hum Pathol 42:1873–1877 21663937 10.1016/j.humpath.2011.02.004

[11] Lai W-A, Hang J-F, Liu C-H et al (2020) PAX8 expression in anaplastic thyroid carcinoma is less than those reported in early studies: a multi-institutional study of 182 cases using the monoclonal antibody MRQ-50. Virchows Arch 476:431–437 31732814 10.1007/s00428-019-02708-4

[12] WHO Classification of Tumors, 5th edition, Endocrine Tumors, IARC, Lyon, 2024 (online)

[13] Lloyd RV, Osamura RY, Kloppel G, Rosai J (2017) WHO classification of tumors of endocrine organs, 4th Edition. IARC, Lyon

[14] Nishiyama RH, Dunn EL, Thompson NW (1972) Anaplastic spindle cell and giant cell tumors of the thyroid g land. Cancer 30:113–127 5040735 10.1002/1097-0142(197207)30:1<113::aid-cncr2820300118>3.0.co;2-e

[15] Aldinger KA, Samaan NA, Iabenz M, Stratton Hill C (1978) Anaplastic carcinoma of the thyroid. A review of 84 cases of spindle and giant cell carcinoma of the thyroid. Cancer 41:2267–2275 657091 10.1002/1097-0142(197806)41:6<2267::aid-cncr2820410627>3.0.co;2-7

[16] Carcangiou ML, Steeper T, Zampi G, Rosai J (1985) Anaplastic thyroid carcinoma. A study of 70 cases. Am J Clin Pathol 83:135–158 2578727 10.1093/ajcp/83.2.135

[17] Xu B, Ghossein RA (2023) Advances in thyroid pathology: high grade follicular cell-derived thyroid carcinoma and anaplastic thyroid carcinoma. Adv Anat Pathol 30:3–10 36306188 10.1097/PAP.0000000000000380

[18] Xu B, Fuchs T, Dogan A et al (2000) Dissecting anaplastic thyroid carcinoma: A comprehensive clinical, histologic, immunophenotypic, and molecular study of 360 cases. Thyroid 30:1505–1517 10.1089/thy.2020.0086 PMC7583343 32284020

[19] Lloyd RV, Osamura RY, Kloppel G, Rosai J (2017) WHO classification of tumors of endocrine organs, 4th ed. IARC, Lyon

[20] Chambers MA, Sadow PM, Kerr DA (2022) Squamous differentiation in the thyroid: metaplasia, neoplasia, or bystander? Int J Surg Pathol 30:385–392 34894811 10.1177/10668969211065126 PMC9323721

[21] Wan S-K, Chan JKC, Tang S-K (1996) Paucicellular variant of anaplastic thyroid carcinoma. A mimic of Riedel’s thyroiditis. Am J Clin Pathol 105:388–393 8604680 10.1093/ajcp/105.4.388

[22] Suster DI, Mackinnon AC, Ronen N, Mejbel HA, Harada S, Michal M, Suster S (2024) Inflammatory giant cell carcinoma of the lung: clinicopathologic, immunohistochemical and next generation sequencing study of 14 cases. Am J Surg Pathol 48:1215–1223 38989701 10.1097/PAS.0000000000002285

[23] Kuhn E, Ragazzi M, Ciarrochi A et al (2019) Angiosarcoma and anaplastic carcinoma of the thyroid are two distinct entities: a morphologic, immunohistochemical, and genetic study. Mod Pathol 32:787–798 30723294 10.1038/s41379-018-0199-z

[24] Albores-Saavedra J, Hernandez M, Sanchez-Sosa S, Simpson K, Angeles A, Henson DE (2007) Histologic variants of papillary and follicular carcinomas associated with anaplastic and giant cell carcinomas of the thyroid: an analysis of rhabdoid and thyroglobulin inclusions. Am J Surg Pathol 31:729–736 17460457 10.1097/01.pas.0000213417.00386.74

[25] Feng G, Laskin WB, Chou PM, Lin X (2015) Anaplastic thyroid carcinoma with rhabdoid features. Diagn Cytopathol 43:416–420 25614185 10.1002/dc.23254

[26] Bansal S, Sancheti S, Kaur S, Somal P, Kalra SK, Sali AP (2020) Anaplastic thyroid carcinoma with rhabdoid phenotype: an unusual case and a comprehensive review. Diagn Cytopathol 48:1125–1130 32515545 10.1002/dc.24516

[27] Gomaa W, Marouf A, Alamoudi A, Al-Maghrabi J (2020) SOX2 is a potential novel marker of undifferentiated thyroid carcinomas. Cureus 12:e12102 33489519 10.7759/cureus.12102 PMC7805510

[28] Li H, Xu T, Zhang R, Wang G, Lu Z, Li Z (2020) TMEM158 may serve as a diagnostic biomarker for anaplastic thyroid carcinoma: an integrated bioinformatic analysis. Curr Med Sci 40:1137–1147 33428142 10.1007/s11596-020-2296-8

[29] Seok JY, Astvatsaturyan K, Peralta-Venturina M, Lai J, Fan X (2021) TROP2, 5hmC, and IDHI expression in anaplastic thyroid carcinoma. Int J Surg Pathol 29:368–377 33289434 10.1177/1066896920978597

[30] Haase J, Misiak D, Bauer M et al (2021) IGF2BP1 is the first positive marker for anaplastic thyroid carcinoma diagnosis. Mod Pathol 34:32–41 32719445 10.1038/s41379-020-0630-0 PMC7806508

[31] Okada T, Nakamura T, Watanabe T et al (2014) Coexpression of EpCAM, CD44 variant isoforms and Claudin-7 in anaplastic thyroid carcinoma. PLoS ONE 9:e94487 24727741 10.1371/journal.pone.0094487 PMC3984167

[32] Mneimneh WS, Asa SL (2024) Divergent lineage markers in anaplastic thyroid carcinoma. Am J Surg Pathol 48:230–237 37972932 10.1097/PAS.0000000000002153

[33] Subbiah V, Kreitman RJ, Weinberg ZA et al (2018) Dabrafenib and trametinib treatment in patients with locally advanced or metastatic BRAF V600e mutant anaplastic thyroid carcinoma. J Clin Oncol 36:7–13 29072975 10.1200/JCO.2017.73.6785 PMC5791845

[34] Cabanillas ME, Dadu R, Ferrarotto R et al (2024) Anti-programed cell death ligand 1 plus targeted therapy in anaplastic thyroid carcinoma: a non-randomized clinical trial. JAMA Oncol 10:1672–1680 39446377 10.1001/jamaoncol.2024.4729 PMC11581602

[35] Mohammed S, Mettman D, Breier AH, Patel V, Garcia-Touza M (2025) Review of genomic drivers of thyroid cancer and their clinical implications. Genes 17(1):36 41595456 10.3390/genes17010036 PMC12840629

[36] Wilson NW, Pambakian H, Richardson TC, Stokoe MR, Makin CA, Heyderman E (1986) Epithelial makers in thyroid carcinoma: an immunoperoxidase study. Histopathology 10:815–829 2428725 10.1111/j.1365-2559.1986.tb02580.x

[37] Hurliman J, Gardiol D, Csazziga B (1987) Immunohistology of anaplastic thyroid carcinoma. A study of 43 cases. Histopathology 11:567–580 2442086 10.1111/j.1365-2559.1987.tb02667.x

[38] Ordonez NG, El-Naggar AK, Hickey RC, Samaan NA (1991) Anaplastic thyroid carcinoma. Immunocytochemical study of 32 cases. Am J Clin Pathol 96:15–24 1712540 10.1093/ajcp/96.1.15

[39] Miettinen M, Franssila KO (2000) Variable expression of keratins and nearly uniform lack of thyroid transcription factor 1 in thyroid anaplastic carcinoma. Hum Pathol 31:1130–1145 10.1053/hupa.2000.16667 11014583

[40] Nonaka D, Tang Y, Chiriboga L, Rivera M, Ghossein R (2008) Diagnostic utility of thyroid transcription factors PAX8 and TTF-2 (FoxE1) in thyroid epithelial neoplasms. Mod Pathol 21:192–200 18084247 10.1038/modpathol.3801002

[41] Bishop JA, Sharma R, Westra WH (2011) PAX8 immunostaining of anaplastic thyroid carcinoma: a reliable means of discerning thyroid origin from undifferentiated tumors of the head and neck. Hum Pathol 42:1873–1877 21663937 10.1016/j.humpath.2011.02.004

[42] Lai W-A, hang J-F, Liu C-Y et al (2020) PAX8 expression in anaplastic thyroid carcinoma is less than those reported in early studies: a multi-institutional study of 182 cases using the monoclonal antibody MRQ-50. Virchows Arch 476:431–437 31732814 10.1007/s00428-019-02708-4

[43] Vennapusa B, Fischer EG, Wick MR, Cerilli LA (2011) CD10 immunoreactivity in sarcomatoid carcinomas. Comparison with true sarcomas. Appl Immunohistochem Mol Morphol 19:408–412 21908981 10.1097/PAI.0b013e3182111be5

[44] Ogawa H, Iwaya K, Izumi M et al (2002) Expression of CD10 by stromal cells during colorectal tumor development. Hum Pathol 33:806–811 12203213 10.1053/hupa.2002.125773

[45] Huang WB, Zhou XJ, Chen JY et al (2005) CD10 positive stromal cells in gastric carcinoma: correlation with invasion and metastases. Jpn J Clin Oncol 35:245–250 15886270 10.1093/jjco/hyi076

[46] Montemayer-Garcia C, Hardin H, Shawn H, Guo et al. (2013) The role of epithelial-mesenchymal transition markers in thyroid carcinoma progression. Endocr Pathol 24:206–212 10.1007/s12022-013-9272-9 PMC3875396 24126800

[47] Nazakawa T, Kondo T, Vuong H et al (2018) High expression of CD10 in anaplastic thyroid carcinomas. Histopathology 73:492–499 29791034 10.1111/his.13657

[48] Yu W, Wang Y, Jiang Y, Zhang W, Li Y (2017) Distinct immunophenotypes and prognostic factors in renal cell carcinoma with sarcomatoid differentiation: a systematic study of 19 immunohistochemical markers in 42 cases. BMC Cancer 17:293 28449664 10.1186/s12885-017-3275-8 PMC5408832

[49] Deniz K, Cohan G, Okten T (2012) Anti-CD10 (56C6) expression in soft tissue sarcomas. Pathol Res Pract 208:281–285 22464152 10.1016/j.prp.2012.02.002

[50] Deeken-Draisey A, Yang GY, Gao J et al (2018) Anaplastic thyroid carcinoma: an epidemiologic, histologic, immunohistochemical, and molecular single-institution study. Hum Pathol 82:140–148 30075157 10.1016/j.humpath.2018.07.027

