# Supplementary material for: Anaplastic thyroid carcinoma: Clinicopathologic and immunohistochemical study of 144 cases with special emphasis on the spectrum of histologic features
Source: Virchows Arch. 2026 Feb 27;488(5):1111–24. doi: 10.1007/s00428-026-04456-8 (PMC13176023; doi:10.1007/s00428-026-04456-8)
Supplement: Supplementary file 1 — Supplementary file1 (DOCX 14 KB) [file 428_2026_4456_MOESM1_ESM.docx]

Supplementary Table 1: Distribution of cases by years and institution in 144 patients with ATC.

| **Source** | **1990-2000** | **2000-2020** | **Total** |
| --- | --- | --- | --- |
| Medical College of Wisconsin | 12 | 14 | 26 |
| Biopticka Lab, Plsen | 9 | 23 | 32 |
| University of Alabama at Birmigham | 15 | 22 | 37 |
| Massachusetts General Hospital | 10 | 25 | 35 |
| Personal Consults | 2 | 12 | 14 |
