# Supplementary material for: Anaplastic thyroid carcinoma: Clinicopathologic and immunohistochemical study of 144 cases with special emphasis on the spectrum of histologic features
Source: Virchows Arch. 2026 Feb 27;488(5):1111–24. doi: 10.1007/s00428-026-04456-8 (PMC13176023; doi:10.1007/s00428-026-04456-8)
Supplement: Supplementary file 2 — Supplementary file2 (DOCX 55 KB) [file 428_2026_4456_MOESM2_ESM.docx]

Supplementary Table 2 – Clinical, pathological, and immunohistochemical features in 144 cases of anaplastic carcinoma of the thyroid.

| No. | Sex/Age | Histology | Associated  Conditions | TTF1 | PAX8 | P40 | AE1/3 | CAM5.2 | CD10 | MIB |
| --- | --- | --- | --- | --- | --- | --- | --- | --- | --- | --- |
| 1 | F/64 | Epithelioid with clear cells and focal squamous differentiation | PTC + Hashimoto | 0 | 0 | 0 | +++ | +++ | + | 60% |
| 2 | F/49 | Epithelioid with osteoclastic giant cells | FVPTC | 0 | 0 | 0 | 0 |  | ++ | 50% |
| 3 | F/57 | Mixed spindle/epithelioid | N/A | 0 | 0 | 0 | 0 | 0 | +++ | 70% |
| 4 | F/82 | Spindle/pleomorphic with focal squamous differentiation | N/A | 0 | +++ | +++ | +++ | 0 | +++ | 60% |
| 5 | M/68 | Spindle/pleomorphic | FVPTC | 0 | 0 | 0 | 0 | 0 | +++ | 50% |
| 6 | F/81 | Epithelioid, “inflammatory”, with squamous differentiation | PTC, oncocytic | 0 | +++ | 0 | +++ | +++ | +++ | 50% |
| 7 | M/58 | Epithelioid with squamous differentiation | FTC + Hashimoto | 0 | +++ | +++ | +++ | +++ | +++ | 50% |
| 8 | M/56 | Mixed spindle/epithelioid with metaplastic bone | FTC. | 0 | 0 | 0 | + | + | ++ | 60% |
| 9 | F/48 | Spindle/pleomorphic | Oncocytic FTC | 0 | 0 | 0 | ++ | + | +++ | 30% |
| 10 | F/65 | Spindle/pleomorphic | N/A | 0 | 0 | 0 | + | + | +++ | 50% |
| 11 | F/60 | Spindle, low-grade with sclerosis + cartilage and pseudoangiosarcomatous areas | Oncocytic FTC | 0 | 0 | 0 | 0 | 0 | ++ | 50% |
| 12 | F/52 | Spindle/pleomorphic with abundant osteoclastic giant cells and foci of squamous differentiation. | Nodular thyroid disease | 0 | 0 | + | 0 | 0 |  | 50% |
| 13 | M/59 | Epithelioid with clear cells and foci of squamous differentiation. | Oncocytic FTC. | 0 | 0 | 0 | + | + | ++ | 50% |
| 14 | M/82 | Mixed spindle/epithelioid with stromal sclerosis, focal pseudoangiosarcomatous areas, and osseous metaplasia. | Nodular thyroid disease | 0 | 0 | 0 | 0 | 0 | ++ | 40% |
| 15 | F/63 | Mixed epithelioid/spindle with paucicellular areas | Nodular thyroid disease + Hashimoto | 0 | ++ | +++ | +++ | +++ | +++ | 60% |
| 16 | M/60 | Mixed epithelioid and spindle with squamous differentiation. | Oncocytic FTC | 0 | +++ | +++ | +++ | +++ | +++ | 30% |
| 17 | M/72 | Epithelioid with clear cells and squamous differentiation | Oncocytic FTC | 0 | +++ | 0 | +++ | +++ | + | 30% |
| 18 | F/75 | Epithelioid with clear cells and squamous differentiation | PTC | + | +++ | 0 | +++ | +++ | O | -- |
| 19 | F/59 | Epithelioid, “inflammatory” | N/A | 0 | +++ | 0 | ++ | ++ | +++ | 50% |
| 20 | M/68 | Epithelioid with squamous differentiation | Oncocytic FTC | 0 | +++ | +++ | +++ | 0 | +++ | 50% |
| 21 | F/63 | Mixed epithelioid/spindle with pseudoangiosarcomatous areas. | N/A | 0 | 0 | 0 | 0 | 0 | +++ | 20% |
| 22 | F/58 | Spindle/pleomorphic with squamous differentiation | Tall cell PTC | 0 | 0 | + | 0 | 0 | ++ | 20% |
| 23 | M/67 | Spindle/pleomorphic with squamous differentiation | PTC |  |  |  |  |  |  |  |
| 24 | M/49 | Epithelioid, clear cell with squamous differentiation | PDTC | 0 | 0 | +++ | +++ | ++ | +++ | 60% |
| 25 | M/72 | Mixed spindle/epithelioid | Nodular thyroid disease | 0 | 0 | 0 | 0 | 0 | +++ | 20% |
| 26 | M/79 | Mixed spindle/epithelioid | N/A | 0 | 0 |  | + | + | -- | 50% |
| 27 | M/48 | Spindle, low-grade, with paucicellular areas. | Nodular thyroid disease | 0 | 0 | ++ | 0 | ++ | +++ | 60% |
| 28 | F/54 | Spindle/pleomorphic with pseudoangiosarcomatous and paucicellular areas. | FVPTC | 0 | 0 | 0 | 0 | 0 | ++ | 40% |
| 29 | F/55 | Spindle/pleomorphic with squamous differentiation and pseudoangiosarcomatous areas | PTC | 0 | +++ | +++ | +++ | 0 | +++ | 20% |
| 30 | F/57 | Epithelioid with paucicellular areas. | Oncocytic PTC | 0 | 0 | 0 | ++ | + | ++ | 50% |
| 31 | F/82 | Spindle/pleomorphic with paucicellular areas. | PDTC | 0 | 0 | 0 | + | 0 | + | 30% |
| 32 | M/76 | Epithelioid with squamous differentiation | Oncocytic PTC | 0 | 0 | 0 | + | 0 | ++ | 40% |
| 33 | M/73 | Epithelioid, “inflammatory” | Oncocytic PTC + Hashimoto |  |  |  |  |  |  |  |
| 34 | M/68 | Epithelioid with clear cells | N/A | 0 | 0 | 0 | 0 | 0 | +++ | -- |
| 35 | F/65 | Spindle, low-grade fascicular | N/A |  |  |  |  |  |  |  |
| 36 | F/74 | Spindle/pleomorphic with osteoclastic giant cells | N/A | 0 | 0 | 0 | 0 | 0 | + | 40% |
| 37 | F/54 | Epithelioid, inflammatory, with rhabdoid cells | N/A | 0 | +++ | 0 | +++ | ++ | +++ | 60% |
| 38 | F/56 | Epithelioid | Nodular thyroid disease | 0 | 0 | 0 | + | 0 | +++ | 30% |
| 39 | F/68 | Epithelioid with squamous differentiation | Nodular thyroid disease | 0 | 0 | +++ | +++ | 0 | ++ | 40% |
| 40 | M/64 | Epithelioid, inflammatory | PTC | 0 | 0 | 0 | ++ | ++ | +++ | 30% |
| 41 | M/71 | Epithelioid, inflammatory | N/A | 0 | 0 | 0 | 0 | 0 | 0 | 60% |
| 42 | F/70 | Spindle, low-grade fascicular | N/A | 0 | 0 | 0 | 0 | 0 | +++ | 60% |
| 43 | F/68 | Spindle/pleomorphic with osteoclastic giant cells | N/A | 0 | 0 | 0 | + | 0 | +++ | 60% |
| 44 | F/64 | Epithelioid | N/A | 0 | ++ | 0 | ++ | 0 | ++ | 70% |
| 45 | M/56 | Epithelioid, paucicellular with pseudoangiosarcomatous foci | FTC | 0 | 0 | 0 | 0 | 0 | +++ | 70% |
| 46 | M/71 | Epithelioid with clear cells | PDTC | 0 | 0 | 0 | 0 | 0 | -- | 70% |
| 47 | F/56 | Epithelioid with pseudoangiosarcomatous areas | N/A | 0 | +++ | +++ | +++ | +++ | ++ | 80% |
| 48 | F/53 | Epithelioid | N/A | 0 | 0 | 0 | 0 | 0 | + | 70% |
| 49 | F/64 | Epithelioid | FTC | 0 | 0 | 0 | 0 | 0 | +++ | -- |
| 50 | M/74 | Epithelioid with pseudoangiosarcomatous areas | Oncocytic FTC | 0 | 0 | 0 | + | 0 | +++ | 80% |
| 51 | M/49 | Epithelioid with squamous differentiation | FTC | 0 | +++ | 0 | +++ | 0 | +++ | -- |
| 52 | M/67 | Epithelioid with abundant osteoclastic giant cells | N/A |  |  |  |  |  |  |  |
| 53 | F/53 | Mixed spindle/epithelioid | Nodular thyroid disease | 0 | 0 | 0 | + | 0 | ++ | 50% |
| 54 | M/63 | Epithelioid | N/A | 0 | 0 | 0 | + | + | +++ | 80% |
| 55 | F/54 | Spindle, low-grade fascicular | FTC | 0 | 0 | 0 | 0 | 0 | +++ | 70% |
| 56 | F/64 | Mixed epithelioid/spindle, with inflammatory features | N/A | 0 | 0 | 0 | 0 | 0 | 0 | 0 - |
| 57 | F/70 | Spindle/pleomorphic | N/A | 0 | + | 0 | +++ | ++ | +++ | 50% |
| 58 | M/65 | Epithelioid with paucicellular areas | FVPTC |  |  |  |  |  |  |  |
| 59 | M/49 | Mixed spindle/epithelioid with osteoclastic giant cells | Nodular thyroid disease |  |  |  |  |  |  |  |
| 60 | M/56 | Epithelioid, inflammatory | FTC | 0 | +++ | 0 | 0 | 0 | +++ | 20% |
| 61 | F/63 | Epithelioid with rhabdoid cells | Nodular thyroid disease + Hashimoto |  |  |  |  |  |  |  |
| 62 | F/64 | Spindle/pleomorphic with squamous differentiation | Nodular thyroid disease | 0 | +++ | +++ | +++ | +++ | +++ | 20% |
| 63 | F/58 | Mixed spindle/epithelioid, with pseudoangiosarcomatous areas | Nodular thyroid disease | 0 | 0 | 0 | 0 |  | +++ | 60% |
| 64 | M/64 | Epithelioid with clear cells, squamous differentiation, and pseudoangiosarcomatous areas | FTC | 0 | 0 | 0 | +++ | +++ | +++ | -- |
| 65 | M/58 | Spindle, low-grade fascicular | FTC | 0 | 0 | 0 | 0 | 0 | +++ | 20% |
| 66 | F/64 | Mixed epithelioid/spindle with paucicellular areas and sclerosis | Oncocytic FTC | 0 | ++ | 0 | +++ | 0 | +++ | 20% |
| 67 | M/59 | Spindle/pleomorphic with squamous differentiation | PTC | 0* | 0 | +++ | +++ | 0 | +++ | 20% |
| 68 | F/60 | Mixed spindle/epithelioid | Oncocytic FTC | 0 | 0 | 0 | +++ | 0 | ++ | 30% |
| 69 | F/65 | Mixed spindle and epithelioid with paucicellular areas. | FTC | 0 | 0 | 0 | 0 | 0 | +++ | 50% |
| 70 | F/70 | Epithelioid with squamous differentiation and paucicellular areas with sclerosis | Nodular thyroid disease | 0 | +++ | +++ | +++ | +++ | ++ | 50% |
| 71 | M/53 | Epithelioid with clear cells | FVPTC | 0 | +++ | 0 | +++ | 0 | +++ | 30% |
| 72 | M/62 | Epithelioid with osteoclastic giant cells | FVPTC | 0 | 0 | 0 | 0 | 0 | + | 50% |
| 73 | M/59 | Epithelioid | Nodular thyroid disease | 0 | 0 | 0 | 0 | 0 | ++ | 40% |
| 74 | M/63 | Mixed spindle/epithelioid with osteoclastic giant cells | FVPTC | 0 | 0 | 0 | 0 | + | +++ | 50% |
| 75 | F/64 | Spindle/pleomorphic with squamous differentiation | Nodular thyroid disease | 0 | 0 | +++ | +++ | +++ | +++ | 40% |
| 76 | F/58 | Spindle/pleomorphic | Nodular thyroid disease | 0 | 0 | 0 | 0 | 0 | +++ | -- |
| 77 | F/80 | Spindle, low-grade fascicular with paucicellular areas. | FVPTC | 0 | 0 | 0 | +++ | + | + | 20% |
| 78 | M/52 | Epithelioid, with foci of squamous differentiation | FTC | 0 | 0 | 0 | + | 0 | 0 | 40% |
| 79 | M/63 | Epithelioid with foci of squamous differentiation | Nodular thyroid disease | ++ | +++ | 0 | +++ | +++ | 0 | 40% |
| 80 | F/67 | Spindle, low-grade fascicular | N/A | 0 | 0 | 0 | + | 0 | +++ | 60% |
| 81 | F/59 | Epithelioid with squamous differentiation | FVPTC |  |  |  |  |  |  |  |
| 82 | M/72 | Mixed spindle/epithelioid | FVPTC | 0 | 0 | 0 | 0 | 0 | +++ | 50% |
| 83 | M/59 | Spindle/pleomorphic | Nodular thyroid disease | 0 | +++ | 0 | + | + | ++ | 50% |
| 84 | M/62 | Epithelioid | Nodular thyroid disease | 0 | 0 | 0 | 0 | 0 | ++ | 20% |
| 85 | F/57 | Epithelioid, inflammatory | N/A | 0 | 0 | 0 | +++ | +++ | +++ | 50% |
| 86 | F/73 | Epithelioid with squamous differentiation | N/A | 0 | +++ | 0 | +++ | ++ | ++ | 50% |
| 87 | F/58 | Spindle/pleomorphic | Nodular thyroid disease | 0 | ++ | 0 | 0 | + | +++ | 30% |
| 88 | M/46 | Epithelioid with focal osseous metaplasia | N/A | 0 | 0 | 0 | 0 | 0 | ++ | 30% |
| 89 | M/63 | Spindle, low-grade fascicular | N/A | 0 | 0 | 0 | + | + | +++ | 20% |
| 90 | M/49 | Spindle/pleomorphic with squamous differentiation | N/A | 0 | 0 | 0 | + | + | +++ | 50% |
| 91 | F/52 | Spindle/pleomorphic | N/A | 0 |  | 0 | ++ | + | ++ | 20% |
| 92 | F/59 | Spindle/pleomorphic | N/A | 0 | 0 | +++ | + | 0 | ++ | 30% |
| 93 | F/64 | Spindle/pleomorphic with squamous differentiation | N/A |  |  |  |  |  |  |  |
| 94 | M/56 | Mixed spindle/epithelioid with squamous differentiation | N/A | 0 | 0 | +++ | +++ | 0 | + | 30% |
| 95 | M/65 | Spindle/pleomorphic | PTC |  |  |  |  |  |  |  |
| 96 | F/60 | Spindle/pleomorphic with squamous differentiation. | Nodular thyroid disease |  |  |  |  |  |  |  |
| 97 | F/54 | Spindle, low-grade fascicular | N/A | 0 | 0 | 0 | 0 | 0 | 0 | 20% |
| 98 | F/70 | Spindle/pleomorphic with squamous differentiation and sclerotic paucicellular areas | N/A | 0 | 0 | 0 | 0 | + | +++ | 30% |
| 99 | M/51 | Mixed epithelioid/spindle with rhabdoid cells. | Nodular thyroid disease | 0 | 0 | 0 | 0 | 0 | +++ | N/A |
| 100 | F/63 | Mixed epithelioid/spindle | Nodular thyroid disease | 0 | 0 | 0 | N/A | ++ | +++ | N/A |
| 101 | F/50 | Spindle/pleomorphic | Nodular thyroid disease | 0 | 0 | 0 | + | +++ | +++ | 60% |
| 102 | F/67 | Epithelioid, clear cell, with squamous differentiation | FVPTC |  |  |  |  |  |  |  |
| 103 | M/69 | Epithelioid, clear cell, with focal rhabdoid features and foci of squamous differentiation | FTC | 0 | ++ | 0 | ++ | ++ | +++ | N/R  N/R |
| 104 | M/70 | Epithelioid, inflammatory with rhabdoid features | Nodular thyroid disease + Hashimoto |  |  |  |  |  |  |  |
| 105 | M/64 | Mixed epithelioid/spindle with foci of squamous differentiation and pseudoangiosarcomatous areas | FTC |  |  |  |  |  |  |  |
| 106 | F/58 | Epithelioid, with focal rhabdoid cells | N/A | 0 | + | 0 | ++ | + | 0 | N/R |
| 107 | F/59 | Epithelioid with rhabdoid cells | N/A |  |  |  |  |  |  |  |
| 108 | F/49 | Spindle/pleomorphic with sclerotic paucicellular areas | FTC | 0 | 0 | 0 | 0 | 0 | +++ | N/R |
| 109 | M/63 | Epithelioid | N/A |  |  |  |  |  |  |  |
| 110 | M/59 | Spindle/pleomorphic with areas of squamous differentiation | PTC | + | + | +++ | ++ | +++ | + | 50% |
| 111 | F/50 | Epithelioid with metaplastic bone | N/A | N/A | 0 | N/A | 0 | N/A | +++ | N/R |
| 112 | F/62 | Mixed spindle/epithelioid | Nodular thyroid disease | 0 | 0 | 0 | 0 |  | +++ | N/R |
| 113 | M/65 | Spindle, low-grade with paucicellular areas | N/A |  |  |  |  |  |  |  |
| 114 | F/64 | Epithelioid, inflammatory, with focal rhabdoid cells | PTC |  |  |  |  |  |  |  |
| 115 | M/58 | Spindle/pleomorphic with foci of squamous differentiation and metaplastic bone | PTC |  |  |  |  |  |  |  |
| 116 | F/60 | Epithelioid with squamous differentiation | Oncocytic PTC |  |  |  |  |  |  |  |
| 117 | F/67 | Epithelioid | Oncocytic PTC |  |  |  |  |  |  |  |
| 118 | M/61 | Epithelioid, inflammatory with pseudoangiosarcomatous areas | PDTC |  |  |  |  |  |  |  |
| 119 | M/58 | Spindle, low-grade paucicellular, with foci of squamous differentiation | Nodular thyroid disease |  |  |  |  |  |  |  |
| 120 | F/64 | Epithelioid | N/A |  |  |  |  |  |  |  |
| 121 | F/56 | Epithelioid | Oncocytic FTC |  |  |  |  |  |  |  |
| 122 | M/64 | Spindle/pleomorphic with foci of squamous differentiation | PTC |  |  |  |  |  |  |  |
| 123 | F/52 | Epithelioid with squamous differentiation | Oncocytic PTC |  |  |  |  |  |  |  |
| 124 | F/47 | Spindle/pleomorphic with squamous differentiation | N/A | 0 | 0 | ++ | ++ | ++ | +++ | 60% |
| 125 | M/58 | Epithelioid with clear cells and focal metaplastic bone | FTC | 0 | ++ | 0 | ++ | 0 | + | 50% |
| 126 | M/64 | Mixed spindle/epithelioid | FTC | 0 | 0 | 0 | + | 0 | +++ | 50% |
| 127 | F/67 | Epithelioid, clear cell with focal paucicellular areas. | Oncocytic FTC | 0 | 0 | 0 | 0 | 0 | +++ | 40% |
| 128 | F/58 | Epithelioid, inflammatory | N/A |  |  |  |  |  |  |  |
| 129 | M/75 | Mixed epithelioid/spindle with squamous differentiation | PTC |  |  |  |  |  |  |  |
| 130 | M/60 | Spindle/pleomorphic | FTC |  |  |  |  |  |  |  |
| 131 | F/58 | Epithelioid with foci of squamous differentiation | Oncocytic PTC | 0 | ++ | 0 | ++ | 0 | +++ | 30% |
| 132 | M/49 | Epithelioid, inflammatory | N/A |  |  |  |  |  |  |  |
| 133 | M/65 | Epithelioid, with rhabdoid cells | Oncocytic PTC | 0 | ++ | ++ | ++ | 0 | +++ | 60% |
| 134 | M/53 | Epithelioid | PTC. | 0 | + | 0 | 0 | 0 | +++ | 40% |
| 135 | F/50 | Epithelioid, with squamous differentiation | N/A | ++ | ++ | 0 | +++ | + | + | 30% |
| 136 | F/61 | Epithelioid, clear cell with squamous differentiation | FVPTC | 0 | ++ | +++ | ++ | + | +++ | 60% |
| 137 | F/67 | Epithelioid, inflammatory | N/A | ++ | ++ | 0 | +++ | + | +++ | 50% |
| 138 | M/58 | Mixed epithelioid/spindle with sclerosis | Nodular thyroid disease |  |  |  |  |  |  |  |
| 139 | M/51 | Mixed epithelioid/spindle with prominent clear cell areas | Nodular thyroid disease |  |  |  |  |  |  |  |
| 140 | F/57 | Spindle, low-grade fascicular with paucicellular areas of stromal sclerosis | Nodular thyroid disease | 0 | 0 | 0 | 0 | 0 | +++ | 80% |
| 141 | F/64 | Epithelioid, with areas of squamous differentiation | Insular CA |  |  |  |  |  |  |  |
| 142 | M/67 | Epithelioid | N/A |  |  |  |  |  |  |  |
| 143 | F/57 | Epithelioid, inflammatory with clear cells | Nodular thyroid disease | 0 | +++ | 0 | 0 | + | +++ | N/A |
| 144 | M/51 | Epithelioid with squamous differentiation and paucicellular areas | Oncocytic FTC |  |  |  |  |  |  |  |

PTC: papillary thyroid carcinoma; FTC: follicular thyroid carcinoma; FVPTC: follicular variant of papillary thyroid carcinoma; PDTC: poorly differentiated thyroid carcinoma; N/A: none available, M: male, F: female, CA: carcinoma, N/R: not reported
